# Physiological predictors of exercise-induced increases in circulating BDNF isoforms and net cerebral lactate exchange in healthy adults

**DOI:** 10.3389/fphys.2026.1862716

**Published:** 2026-07-06

**Authors:** Olga Tarassova, Yiwen Jiang, Helena Wallin, Julia Röja, Marjan Pontén, Jonna Nilsson, Marcus Moberg, Maria M Ekblom

**Affiliations:** 1 Department of Health Sciences, The Swedish School of Sport and Health Sciences (GIH), Stockholm, Sweden; 2 Department of Physiology, Nutrition, and Biomechanics, The Swedish School of Sport and Health Sciences (GIH), Stockholm, Sweden; 3 Department of Laboratory Medicine, Division of Clinical Physiology, Karolinska Institutet, Stockholm, Sweden; 4 Department of Clinical Physiology, Karolinska University Hospital, Stockholm, Sweden

**Keywords:** adrenaline, arterial-venous difference, cortisol, exercise intensity, lactate, noradrenaline, proBDNF, serum mature BDNF

## Abstract

We previously showed an acute intensity-dependent increase in circulating brain-derived neurotrophic factor (BDNF) after aerobic exercise, without a concurrent increase in its release from the brain. Several exercise-related factors have been suggested to influence BDNF signalling and lactate dynamics, implicated in neuroplastic processes. We investigated whether pre-exercise levels and exercise-induced changes in platelet count, lactate, cortisol and noradrenaline predict changes in forearm venous serum mature BDNF (mBDNF-S) and its precursor proBDNF. As lactate may stimulate neuronal BDNF expression, net cerebral lactate exchange (LAC-AV-B) and its correlates, including mBDNF-S and proBDNF, were investigated. An association with the proportion of fast-twitch muscle fibre cross-sectional area (fCSA%-II) and the moderating effect of BDNF genotype were also examined. Healthy, physically fit adults (n=16, 31 ± 6 yrs) cycled for 20 min at 40, 60 and 80% of VO_2_max, separated by 30-min rest. All biomarkers were analysed in blood samples taken before and after cycling at each intensity. Increases in noradrenaline and lactate were related to increases in mBDNF-S, while increases in cortisol were related to increases in proBDNF. LAC-AV-B was related only to increases in circulating lactate. Changes in mBDNF-S were predicted by fCSA%-II, while increases in proBDNF were not. Exploratory analyses also revealed hypothesis-generating observations of a possible pattern of higher proBDNF levels in Val66Val carriers, whereas a trend toward greater increases in LAC-AV-B in Val66Met carriers. Finally, findings raise the possibility that catecholamine-related increases in mBDNF-S and cortisol-related increases in proBDNF may be linked to mechanisms previously associated with synaptic strengthening and pruning, respectively.

## 1 Introduction

Animal studies have demonstrated the role of the mature form of brain-derived neurotrophic factor (mBDNF) and its antagonist precursor proBDNF in neural plasticity (Woo et al., 2005; Park and Poo, 2013). While mBDNF promotes synaptic strengthening and hippocampal long-term potentiation (LTP), proBDNF promotes synaptic pruning and long-term depression (LTD) (Woo et al., 2005). A single exercise bout has been shown to induce intensity-dependent increases in circulating levels of mBDNF and proBDNF in humans (Reycraft et al., 2020; Edman et al., 2024; Tarassova et al., 2025), suggesting that this may represent a mechanism by which exercise benefits brain function. However, results from Tarassova et al. (2025) do not support this suggestion, indicating instead that exercise-induced increases in mBDNF are driven by platelet release, with no release or uptake by the brain. Several exercise-related physiological factors have been suggested to stimulate and interact with BDNF metabolism. These factors include platelet splenic release (Walsh and Tschakovsky, 2018), increased lactate production (Xue et al., 2022), and increased hormonal activity of catecholamines (McMorris, 2016) and cortisol (Gray et al., 2013; Chen et al., 2017). This may potentially explain the observed increases in circulating BDNF.

Human platelets have been suggested to play an important role in brain plasticity, as upon activation, they enable periphery-brain crosstalk by secreting bioactive molecules into the circulation and communicating with neural cells (Leiter and Walker, 2019). Platelets bind substantial amounts of mBDNF and, to some extent, also proBDNF (Le Blanc et al., 2020). However, Le Blanc et al. (2020) showed that platelet activation induced by several agonists triggered the release of mBDNF only, while proBDNF levels remained stable. In our previous work (Tarassova et al., 2025), we demonstrated that the association between changes in platelet count and mBDNF-S, which mainly represent platelet-bound mBDNF (Fujimura et al., 2002), may reflect splenic platelet release in response to exercise. To nuance this finding, the extent of association between changes in mBDNF-S and other exerkines warrants further exploration.

Previous research has identified lactate as an important energy source for the mammal brain (van Hall et al., 2009; Xue et al., 2022). In the brain, lactate is predominantly produced by astrocytes via glycolysis and transferred to neurons in response to increased neuronal activity (Magistretti and Allaman, 2018; Xue et al., 2022). It has also been shown that lactate can be both released and taken up by the brain at rest and during exercise (Overgaard et al., 2012; Hashimoto et al., 2018), as well as released from skeletal muscle in response to exercise (Juel et al., 2004). In animal models, beyond its metabolic role, lactate has been proposed as a potential mediator of exercise-induced brain BDNF production (El Hayek et al., 2019). In humans, lactate has also been proposed to regulate peripheral mBDNF concentrations (Schiffer et al., 2011). A positive correlation has been demonstrated between mBDNF-S and blood lactate levels measured immediately after a graded cycling exercise test (Ferris et al., 2007), but these associations may be confounded by parallel increases in peripheral platelet count. Recent studies investigating the relationship between circulating lactate and BDNF, however, yielded somewhat different results (Edman et al., 2024, Edman et al., 2025; Röja et al., 2025). A greater increase in plasma mBDNF was observed during resistance exercise with concurrent lactate infusion, with no effect on proBDNF (Edman et al., 2024). However, no association between lactate and plasma levels of either mBDNF or proBDNF was observed after aerobic exercise in Edman et al. (2025), whereas in Röja et al. (2025), 1-h lactate infusion at rest elevated plasma levels of proBDNF, but not mBDNF. Such inconsistencies in previous reports regarding the relationship between lactate and BDNF isoforms warrants further investigation.

Physical exercise also activates neuroendocrine pathways of the sympathetic–adrenal–medullary (SAM) system and hypothalamic–pituitary–adrenal (HPA) axis, leading to increased secretion of catecholamines, such as adrenaline and noradrenaline, and glucocorticoids, such as cortisol (Chen et al., 2017). Exercise effects on cortisol have been shown to depend on exercise intensity (Hill et al., 2008; Chen et al., 2017). During exercise, cortisol plays an important role in metabolic adaptations, including glucose production, maintenance of blood pressure, and muscle function (Deuster et al., 1989; Del Corral et al., 1998). Peripheral cortisol can also cross the blood-brain barrier and, in interaction with trophic factors such as BDNF, influence the regulation of neuroplasticity (Gray et al., 2013; Tsimpolis et al., 2024). Furthermore, concurrent increases in peripheral cortisol and BDNF levels in response to exercise have been previously reported (Rojas Vega et al., 2006; Hötting et al., 2016). However, no association between changes in cortisol and BDNF was detected after either cycling exercise (Gustafsson et al., 2009) or 1-h lactate infusion at rest (Röja et al., 2025). Hence, experimental evidence directly linking exercise-induced changes in BDNF and cortisol remains limited and inconclusive.

A single bout of cycling exercise can induce increases in adrenaline and noradrenaline (Dalsgaard et al., 2004; Hashimoto et al., 2018), and the response is exercise-intensity-dependent (Acevedo et al., 2007; Eugster et al., 2022). Unlike cortisol, peripheral catecholamines do not readily cross the blood-brain barrier (Cornford et al., 1982). However, increased circulating adrenaline and noradrenaline can influence executive function (Grego et al., 2004; McMorris, 2016) via activation of β-adrenoceptors on the afferent vagus nerve, which further triggers activation and synaptic communication between the nucleus tractus solitarii and the locus coeruleus (McGaugh et al., 1996; McMorris, 2016). Activation of β-adrenoceptors, as shown in animal studies, can enhance memory consolidation in the hippocampus (Hu et al., 2007) and induce long-term potentiation (Gelinas and Nguyen, 2005). A link between exercise-induced changes in noradrenaline and BDNF, which may facilitate long-term potentiation, has also been suggested (McMorris, 2016). During high-intensity exercise, noradrenaline-activated β-adrenoceptors stimulate activation of the cyclic adenosine monophosphate messenger (cAMP), which, in turn, modulates signalling and trafficking of the BDNF receptor tropomyosin-related kinase B (TrkB) (Ji et al., 2005; McMorris, 2016). Moreover, it has been shown that, by stimulating β-adrenoceptors, adrenaline can affect lactate uptake by active skeletal muscle in mice (Hamann et al., 2001).

BDNF is expressed in the brain (Park and Poo, 2013), cerebrospinal fluid (Li et al., 2009), blood and platelets (Fujimura et al., 2002), as well as in skeletal muscle (Matthews et al., 2009; Máderová et al., 2019). During physical exercise, lactate is predominantly produced by fast-twitch glycolytic (type II) skeletal muscle fibres and taken up by slow-twitch oxidative (type I) muscle fibres (Gladden, 2004; Brooks et al., 2022). Two recent studies in humans have shown that proBDNF, but not mBDNF, is highly expressed in skeletal muscle (Edman et al., 2024; Röja et al., 2025). ProBDNF expression was predominantly observed in type I muscle fibres (Edman et al., 2024) and muscle levels of proBDNF positively correlated with the percentage of type I muscle fibre cross-sectional area (fCSA%) (Röja et al., 2025). Interestingly, muscle proBDNF levels increased after resistance exercise, but showed no additional response to simultaneous lactate infusion during resistance exercise (Edman et al., 2024). Furthermore, muscle proBDNF levels remained unchanged by lactate infusion at rest (Röja et al., 2025). As a plausible explanation, the authors in Röja et al. (2025) suggest that exercise, rather than lactate, stimulates the release of proBDNF from active skeletal muscle, followed by its potential cleavage to mBDNF (Kojima et al., 2019).

Earlier studies have suggested that genetic variation of BDNF, caused by valine (Val) to methionine (Met) substitution at codon 66 (Val66Met) of the BDNF gene, may influence the BDNF response to exercise and aerobic training (Ieraci et al., 2016; Lemos et al., 2016). More specifically, after four months of aerobic training, handgrip exercise-induced increases in mBDNF-S were observed only in BDNF Val66Val carriers (Lemos et al., 2016). In animal models, Val66Met mutant mice showed impaired behavioural and neuroplastic benefits after four weeks of voluntary exercise compared with the Val66Val mice (Ieraci et al., 2016). In humans, Val66Met has been shown to negatively affect intracellular trafficking and activity-dependent secretion of BDNF (Egan et al., 2003). However, a recent study in humans reported that both Val66Val and Val66Met carriers exhibited similar increases in mBDNF-S and lactate levels in response to submaximal graded exercise (Ashcroft et al., 2025). Further research is needed to clarify these effects.

Acute exercise increases circulating mBDNF-S and proBDNF, as well as net cerebral lactate exchange. These responses have been proposed to reflect mechanisms underlying exercise-induced brain adaptations, but the underlying pathways remain unclear. This study aimed to identify physiological factors that may contribute to the exercise-induced changes in these exerkines. We investigated whether pre-exercise levels and/or exercise-induced changes in platelet count, circulating lactate, cortisol and noradrenaline, as well as type II fCSA% (fCSA%-II), can predict exercise-induced changes in mBDNF-S and proBDNF after 20 min of high-intensity cycling exercise. We also examined whether net cerebral lactate exchange in an exercise-intensity-dependent manner and whether they are associated with changes in mBDNF-S and proBDNF, platelet count, circulating lactate, cortisol and noradrenaline. Additionally, we explored whether genetic variants of the BDNF gene moderate the effects of exercise on mBDNF-S, proBDNF and net cerebral lactate exchange.

## 2 Materials and methods

### 2.1 Ethical approval

This study was approved by the Swedish Ethical Review Authority (Dnr 2022-01368-01) and conformed to the standards set forth in the Declaration of Helsinki. All recruited participants received detailed study information and signed a written informed consent form prior to participating.

### 2.2 Participants

The recruitment process, inclusion and exclusion criteria, and screening procedures have been described previously (Tarassova et al., 2025). In brief, 16 healthy, physically fit adults (8 men, 8 women; age 20–40 years; BMI 19–26 kg/m²; VO_2_max 45–60 mL·kg⁻¹·min⁻¹) were included following initial screening, an information meeting and a fitness assessment.

### 2.3 Study design and procedures

#### 2.3.1 Main experimental protocol

As described in Tarassova et al. (2025), this study employed a within-subject, repeated-measures design to assess the effects of different exercise intensities on various biomarkers. The main exercise protocol involved three intensities of cycling exercise: 40%, 60%, and 80% of each participant’s individual VO₂max, performed during the same session in an intensity-increasing order. Each exercise bout lasted 20 minutes and was followed by 30 minutes of seated rest [see **Figure 1** in Tarassova et al. (2025) for more details on study design]. Each participant’s individual VO₂max was determined from the fitness assessment results.

**Figure 1:**
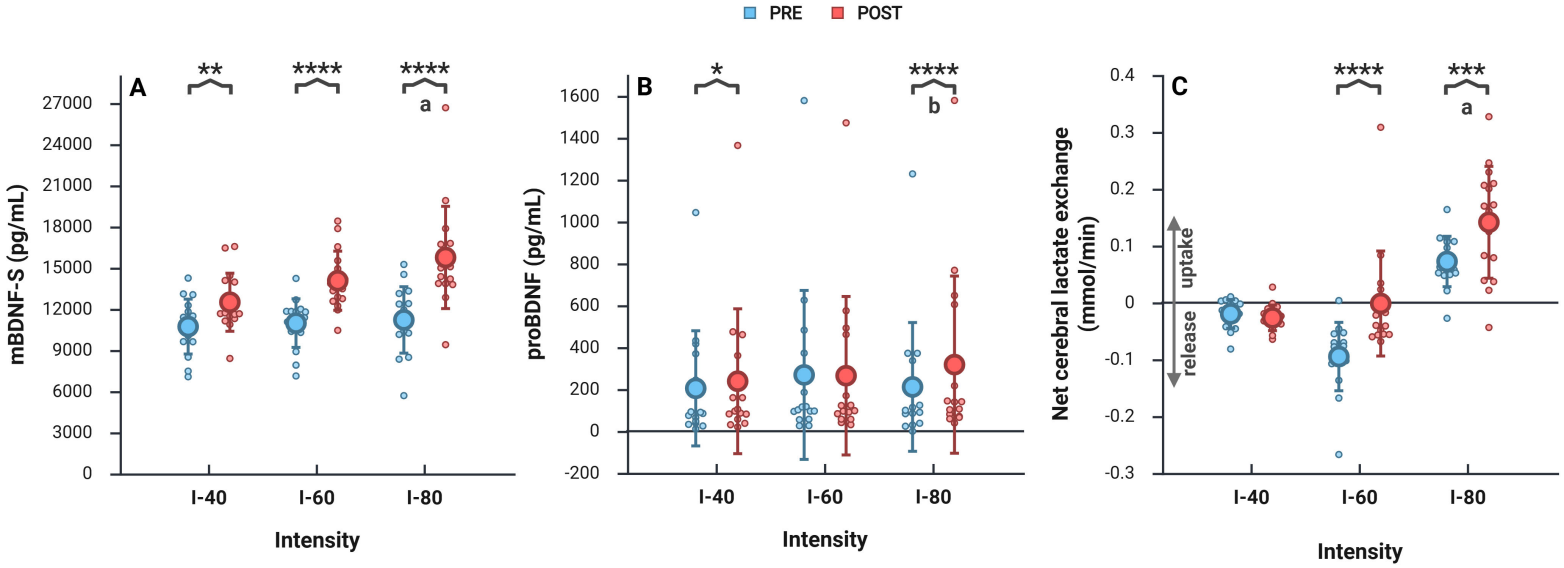
Effects of exercise intensity on forearm venous mBDNF-S, proBDNF and net cerebral lactate exchange. Means (big circles), standard deviations (vertical lines) and individual data points (small circles) of **(A)** forearm venous mBDNF-S (n = 16), **(B)** forearm venous proBDNF (n = 15) and **(C)** brain exchange of lactate (n = 16). Data was analysed using a linear mixed effects model with two fixed effects: time [before (PRE) and after (POST) each intensity level] and intensity [cycling at 40% (I-40), at 60% (I-60) and at 80% (I-80) of individual VO_2_max]. * *p* < 0.05; ** *p* < 0.01; *** *p* < 0.001; **** *p* < 0.0001; a = significant difference from PRE-POST change at I-40; b = significant difference from PRE-POST change at I-60.

Following a familiarization visit, each participant participated in the main experimental session conducted at the Department of Clinical Physiology and the Department of Cardiology at the Karolinska University Hospital. Participants arrived at the hospital at 06:30 AM in an overnight fasted state. Upon arrival, catheters, later used for arterial and venous blood sampling, were inserted into the participants’ left antecubital vein, right brachial artery, right internal jugular vein, and right femoral vein under local anaesthesia and ultrasound guidance. Participants then rested for 15 minutes supine on a hospital bed before the start of the main experimental protocol.

Blood samples (5 mL) were collected simultaneously from all four catheters at 13 time points: at baseline and 60–90 s after 10 min of cycling at each intensity, each cycling bout, and the subsequent 15- and 30-min rest periods. Simultaneously with each blood sample, cerebral and peripheral blood flow velocities were registered using Transcranial Doppler (TCD) ultrasonography for the middle cerebral artery and Doppler ultrasound for the common femoral artery. To ensure practicality, blood samples and concurrent blood flow velocity measurements were obtained with participants in a supine position on a hospital bed. Heart rate and perceived exertion (RPE) were recorded throughout the exercise protocol. After the final blood sample, catheters were removed, and the incisions were cleaned and disinfected. The main session was concluded after confirming that participants were stable and in good health.

Blood sampling procedures involved collecting each blood sample into separate vacutainers: one for plasma (EDTA tube) and one for serum (clot activator tube). Plasma was immediately separated by centrifugation at 3000g for 10 minutes at 4 °C, while serum samples were allowed to clot for at least 10 minutes before centrifugation at 2000g for 10 minutes at 4 °C. Both plasma and serum samples were stored at -80 °C for future analysis. Arterial blood samples were also analysed for platelet count using an automated blood analyser (Sight OLO, S.D. Sight Diagnostics LTD, Israel).

#### 2.3.2 Muscle biopsy session

Within a month after the main experimental session, participants visited the laboratory to donate a muscle biopsy from the vastus lateralis of their right leg. The procedure was performed by trained personnel with the participant in a supine position. Following local anaesthesia and a skin incision approximately 0.5 cm in length, the muscle sample (approximately 100–150 mg) was obtained from the incision using a Bergström needle with applied suction (Evans et al., 1982) and divided into two pieces. Fiber bundles appropriate for histology were oriented perpendicular to the horizontal surface, mounted in O.C.T. embedding medium (Tissue-Tek O.C.T, compound), frozen in isopentane chilled by liquid nitrogen, and stored at -80 °C until cryosectioning.

### 2.4 Blood biomarkers, muscle fibre type composition and BDNF genotype

#### 2.4.1 Blood biomarkers

Platelet count, forearm venous mBDNF-S and plasma proBDNF were earlier analysed in Tarassova et al. (2025). Additionally for this study, arterial plasma lactate (LAC-A), plasma lactate in samples from the jugular vein (LAC-J) and from the femoral vein (LAC-F), as well as arterial serum cortisol (CORT-A), adrenaline (ADR-A) and noradrenaline (NAD-A) were analysed in six out of 13 sample points: before (PRE) and 60–90 s after (POST) 20-min cycling at each of the three workloads: 40% (I-40), 60% (I-60) and 80% (I-80) of participant’s individual VO₂max.

Biomarker concentrations were quantified using target-specific ELISA kits and the manufacturer’s instructions, except for plasma lactate concentrations, which were assessed using a custom-developed laboratory protocol, described in detail in Röja et al. (2025). Forearm venous mBDNF-S concentrations were analysed in duplicates using a Human Free BDNF Immunoassay kit (Quantikine ELISA, DBD00, R&D Systems, Inc., Minneapolis, USA), whereas proBDNF was analysed using the Human Pro-BDNF Development kit (DuoSet ELISA, DY3175, R&D Systems, Inc., Minneapolis, USA). CORT-A was quantified using a Cortisol ELISA kit (Cortisol R2 RC, CO368S, Calbiotech Inc., El Cajon, CA) while ADR-A and NAD-A concentrations were analysed using Adrenaline/noradrenaline ELISA (2-CAT high sensitive ELISA, BA E-5400R, Immusmol SAS, Bordeaux, FR). All BDNF, ADR-A and NAD-A concentrations were expressed in pg/mL, whereas LAC-A was expressed in mmol/l and CORT-A in ng/ml.

Following the procedure detailed in Tarassova et al. (2025), brain exchange of lactate (LAC-AV-B) was quantified as the arterial–venous (a-v) difference in lactate concentration adjusted for changes in blood flow using LAC-A and LAC-J samples. Skeletal muscle exchange of lactate (LAC-AV-M) was measured using LAC-A and LAC-F samples. The LAC-AV-B and LAC-AV-M were expressed in mmol/min.

#### 2.4.2 Muscle fibre type composition

Muscle cross-sections (7 µm) were prepared from the OCT-embedded samples using a cryostat (Leica CM1950), placed on microscope glass slides (VistaVision, VWR International), air-dried for 1h at room temperature, and stored at -80 °C. Immunohistochemical procedures were adapted from previous publications from our laboratory (Horwath et al., 2021). Briefly, unfixed slides were probed with primary antibodies against myosin heavy chain (MyHC) isoform I (BA-F8, 1:200), and dystrophin (MANDYS1, 1:200), purchased from Developmental Studies of Hybridoma Bank (DSHB). The next day, slides were incubated with secondary antibodies (diluted 1:300), all purchased from Alexa Fluor, Invitrogen USA, and mounted with anti-fade fluorescent mounting media (Thermo Fischer Scientific, USA) and a coverslip.

Sections stained for analysis of fibre type composition and fibre cross-sectional area (fCSA) were captured with a widefield fluorescence microscope using a 4x and a 10x objective, respectively (CELENA S; Logos Biosystems, South Korea) and processed with the built-in image analysis software. To determine fibre type composition, captured images were stitched together to reconstruct the whole section using Image J (National Institutes of Health). From this image, the number of each fibre type was manually counted and expressed as a percentage of the total fibre number count, including 535 ± 205 fibres (range 219-948). Fibers stained positive for the BA-F8 antibody were considered type I fibres, whereas fibres stained negative for the BA-F8 antibody were considered type II fibres.

Analysis of fCSA was performed with the semi-automated MyoVision software (Wen et al., 2018). Here, we first performed an in-house validation by manually measuring the fCSA of 400 individual fibres in ImageJ (National Institutes of Health) and comparing the values with those obtained using the software. With an accuracy of 97.0 ± 3.7%, the MyoVision software demonstrated excellent validity relative to manual quantification and was therefore used for the remainder of the study (data not shown). Subsequently, we captured 3–6 images of dystrophin staining from different regions of the cross-section, and each fibre was later identified as either a type I fibre (BA-F8 positive) or a type II fibre (BA-F8 negative) using the overlay image. An average of 89 ± 40 (range 52-200) and 49 ± 18 (range 17-83) fibres was included in the analysis for type I and type II fCSA, respectively. The fCSA%-II was calculated as previously described (Horwath et al., 2021).

#### 2.4.3 BDNF genotyping

Muscle tissues from each participant were genotyped for Val66Met BDNF gene polymorphism (rs6265). The DNA extraction was performed using Qiagen DNeasy^®^ Blood & Tissue kit (QIAGEN AB, Kista, SE) according to the manufacturer’s instructions. Polymerase chain reaction (PCR) amplifications were performed using the Qiagen Taq PCR core kit (QIAGEN AB, Kista, SE) with PCR primers (Sigma) designed according to an earlier reported one-step PCR method (Sheikh et al., 2010). A total volume of 37.2 µl of the PCR mixture used for the amplification reactions contained approximately 25 ng of genomic DNA template, 10 µl of PAR buffer, 2 µl of 2.5 mM deoxyribonucleotide triphosphate (dNTP), 0.5 µl of Taq DNA polymerase, 6 µl of MgCl, and the four primers. The PCR protocol included an initial denaturation for 5 min at 94 °C, followed by 30 cycles of 94 °C for 45 s, 62.5 °C for 60 s, and 72 °C for 60 s, and a final extension at 72 °C for 5 min. Following the addition of EZ load 100bp molecular ruler (BIORAD) and a 1-h DNA gel electrophoresis, the PCR amplicons were stained with SYBR Safe DNA Gel Stain (Invitrogen) for 30 minutes. As a result, the PCR reaction amplified G (val) and A (met) allele-specific amplicons (253 and 201 bp, respectively), whereas the internal control was represented by an amplified 401 bp amplicon.

### 2.5 Statistical analysis

All statistical analyses were performed using RStudio (version 2024.12.1.563) and R programming environment (version 4.5.0), except for an earlier reported *a priori* calculation of the required sample size of 12 participants (Tarassova et al., 2025), which was performed using G*Power (v.3.1.9.6, Franz Faul, Universität Kiel, Germany).

#### 2.5.1 Missing data

In the final dataset used for this study, data were missing completely for proBDNF from one participant (n = 15) and for platelet count from another (n = 15).

#### 2.5.2 Linear mixed-effects models

A linear mixed-effects model was used to assess the main effects of time and exercise intensity, as well as their interactions on all investigated variables. The model included two fixed-effect factors: *time* (PRE and POST) and *intensity* (cycling at I-40, at I-60 and at I-80), while participants were treated as random effects. To facilitate interpretability, the time point PRE and the highest intensity level (I-80) were specified as reference levels so that the main effect of time represents PRE-to-POST change at I-80. To assess whether the BDNF genotype modifies the effect of exercise intensity on the investigated outcomes, a third fixed-effect factor, *BDNF gene* (Val66Met and Val66Val), was included in a separate model. In this model, intensity I-80, time point PRE and Val66Met genotype were defined as reference conditions in the fitted model. The *lmer* function with restricted maximum likelihood (REML) from the *lmerTest* package (version 3.1-3) in R was used to fit linear mixed-effects models. The main effects and interactions were assessed using type III analysis of variance (ANOVA) with denominator degrees of freedom estimated using Satterthwaite’s approximation. To follow up on significant interactions, *post hoc* comparisons were conducted using the default output from the fitted model. Additionally, pairwise comparisons that were missing in the default output from the fitted model were conducted using *difflsmeans* function of the *lmerTest* package. The p-values calculated from the latter comparisons were adjusted for multiple comparisons using false discovery rate (FDR) correction according to the Benjamini–Hochberg procedure. Normal Q–Q plots and the Shapiro-Wilks test for normality of the model residuals were used to assess normality. If violated, log-transformed data were used for statistical analysis. However, for each variable, the untransformed data, expressed as means and standard deviations (SD), were presented in figures with statistical results. Cook’s distance (Di) with a threshold set at Di ≥ 1 was used to detect influential outliers. If the outlier (s) were detected and the model performance improved after their exclusion, the results from the re-fitted model were used instead. For each outcome variable, the percentage of the PRE-POST changes presented in the results section was calculated using untransformed values. The significance level was set at *p* < 0.05. A tendency for a significant difference was set at 0.05 < *p* < 0.1.

The effect of exercise on platelet count and forearm venous mBDNF-S and proBDNF was previously analysed in Tarassova et al. (2025). However, as the present study used a different subset of time points (six out of 13), the data were re-analysed for the selected time points and were presented in the Results.

#### 2.5.3 Multiple regression models

An Elastic Net regression model was used to evaluate associations between the outcomes and predictors, that is, to determine if PRE values of and/or exercise-induced changes in the selected predictors predict exercise-induced changes in the selected outcome.

Given the relatively small sample size and multicollinearity among predictors in this study, an Elastic Net regression model with Leave-One-Out Cross-Validation (LOOCV) was employed (Efron and Tibshirani, 1997; Sylvain and Alain, 2010). This approach combines Lasso (L1) and Ridge (L2) penalties to mitigate the risk of model underfitting or overfitting. All predictors were standardized (centred and scaled). Model hyperparameters α (mixing ratio between L1 and L2 penalties) and λ (overall strength of model regularization) were tuned via grid search over a range of 9 α values evenly spaced within a narrowed range between 0.1 and 0.9 (to avoid model instability associated with extreme values, particularly given the small sample size) and a sequence of 100 λ values. The optimal combination of α and λ minimizing the model Mean Squared Error (MSE) was selected from cross-validation (λ_min_) using the cv.*glmnet* function from the *glmnet* (version 4.1-10) package in R. For the best-performing model (i.e., the one with the lowest MSE), final coefficient estimates (*β*) and model performance metrics, including RMSE, Mean Absolute Error (MAE) and coefficient of determination (R²), which quantifies the proportion of variance in the outcome explained by the model, were computed. Confidence intervals from Elastic Net estimates can be biased and sensitive to data perturbations (Chatterjee and Lahiri, 2011). Therefore, robustness of predictors was assessed through bootstrap resampling with 1000 iterations using the *boot* (version 1.3-31) package in R, and the calculation of the selection stability (π_j_) of the model predictor, defined as the proportion of bootstrap samples in which each predictor had a non-zero coefficient (Meinshausen and Bühlmann, 2010). In each bootstrap iteration, the model was refitted to a resampled dataset, and the optimal α and λ parameters were selected by minimizing the MSE. Predictors with selection proportions ≥ 0.9 were considered stable, values between 0.6 and 0.9 moderately stable and < 0.6 unstable (Meinshausen and Bühlmann, 2010; Hofner et al., 2015; Nogueira et al., 2017). Additional diagnostics of the model performance included: 1) Normal Q-Q plots of residuals to assess normality, 2) Residuals vs. Fitted values plots to evaluate linearity, 3) Spread-Location plots to assess homoscedasticity, and 4) a correlation matrix of predictor variables to inspect potential multicollinearity. Cook’s distance (Di) with a threshold of Di ≥ 1 was used for the detection of influential outliers. If the outlier(s) were detected and the model’s performance metrics improved after their removal, the results from the re-fitted model were employed. Although elastic net regression improves predictive robustness and addresses multicollinearity among predictors through regularization, the identified predictors should be interpreted as associative rather than causal.

#### 2.5.4 Additional correlations

To facilitate discussion of some results in the present study, a Pearson’s correlation coefficient (*r*) between pairs of selected variables was additionally assessed. The significance level was set at p < 0.05.

## 3 Results

### 3.1 Descriptives

Participants’ physical characteristics are described in detail in Tarassova et al. (2025). In the present study, the final dataset included 16 healthy, physically fit adults (**Table 1**).

**Table 1:** Characteristics of the study participants (n = 16).

Characteristic	Mean	SD
Age (years)	31	6
Sex (F/M, %)	50/50	–
Height (cm)	175.1	10.3
Weight (kg)	68.8	9.1
BMI (kg/m^2^)	22.4	1.5
VO_2_max (mL kg^-1^ min^-1^)	54.9	4.1
HR_max_ (bpm)	186	4
Fat (%)	17.9	4.9
Type II muscle fCSA%	36.5	20.2
BDNF genotype (Val66Met/Val66Val, n)	5/11	–

F, Female. M, Male. BMI, body mass index. VO_2_max, maximum rate of oxygen consumption. HR_max_, maximal heart rate. Fat, fat mass as a percentage of total body mass. fCSA%, percentage fibre type cross-sectional area. Val66Met, BDNF Val66Met polymorphism. Val66Val, BDNF Val66Val polymorphism.

### 3.2 Effects of exercise on forearm venous mBDNF-S, proBDNF and net cerebral lactate exchange

For forearm venous mBDNF-S (n = 16; **Figure 1A**), there was a *time × intensity* interaction [*F*(2,74.0) = 3.38, *p* = 0.039]. Increases in mBDNF-S from PRE to POST were observed at all intensities. The mBDNF-S increased by ~17, 28 and 41% at I-40 (*p* = 0.005), I-60 (*p* < 0.001) and I-80 (*p* < 0.001), respectively. The increase at I-80 was greater than at I-40 (*p* = 0.012) but did not differ from the increase shown at I-60.

For log-transformed forearm venous proBDNF (n = 15, **Figure 1B**), a *time × intensity* interaction [*F*(2,69.0) = 5.57, *p* = 0.006] was also significant. The proBDNF increased from PRE to POST by ~17% at I-40 (*p* = 0.017) and by ~50% at I-80 (*p* < 0.001), with no change shown at I-60. The increase at I-80 differed from the non-significant change at I-60 (*p* = 0.001) and showed a trend towards being greater than at I-40 (*p* = 0.080).

For LAC-AV-B (n = 16; **Figure 1C**), there was a *time × intensity* interaction [*F*(2,75.0) = 6.96, *p* = 0.002]. At I-40, no change was observed. Interestingly, at I-60 at PRE, the LAC-AV-B release (negative values below the zero line) occurred, returning to baseline levels at POST (~98%, *p* < 0.001). In contrast, at I-80, a small LAC-AV-B uptake (positive values above the zero line) was shown at PRE. At I-80, the LAC-AV-B uptake increased by ~95% from PRE to POST (*p* = 0.001). The change in LAC-AV-B at I-80 differed from the non-significant change shown at I-40 (*p* = 0.008) but not from the change at I-60.

For log-transformed LAC-AV-M (n = 16; data not illustrated), a *time × intensity* interaction [*F*(2,75.0) = 11.62, *p* < 0.001] was also found. At I-80, there was a release of LAC-AV-M (*β* = 0.50, SE = 0.09, *p* < 0.001). However, no change was shown at either I-40 or at I-60.

### 3.3 Effects of exercise intensity on platelet count, circulating lactate, cortisol, adrenaline and noradrenaline

**Figure 2:**
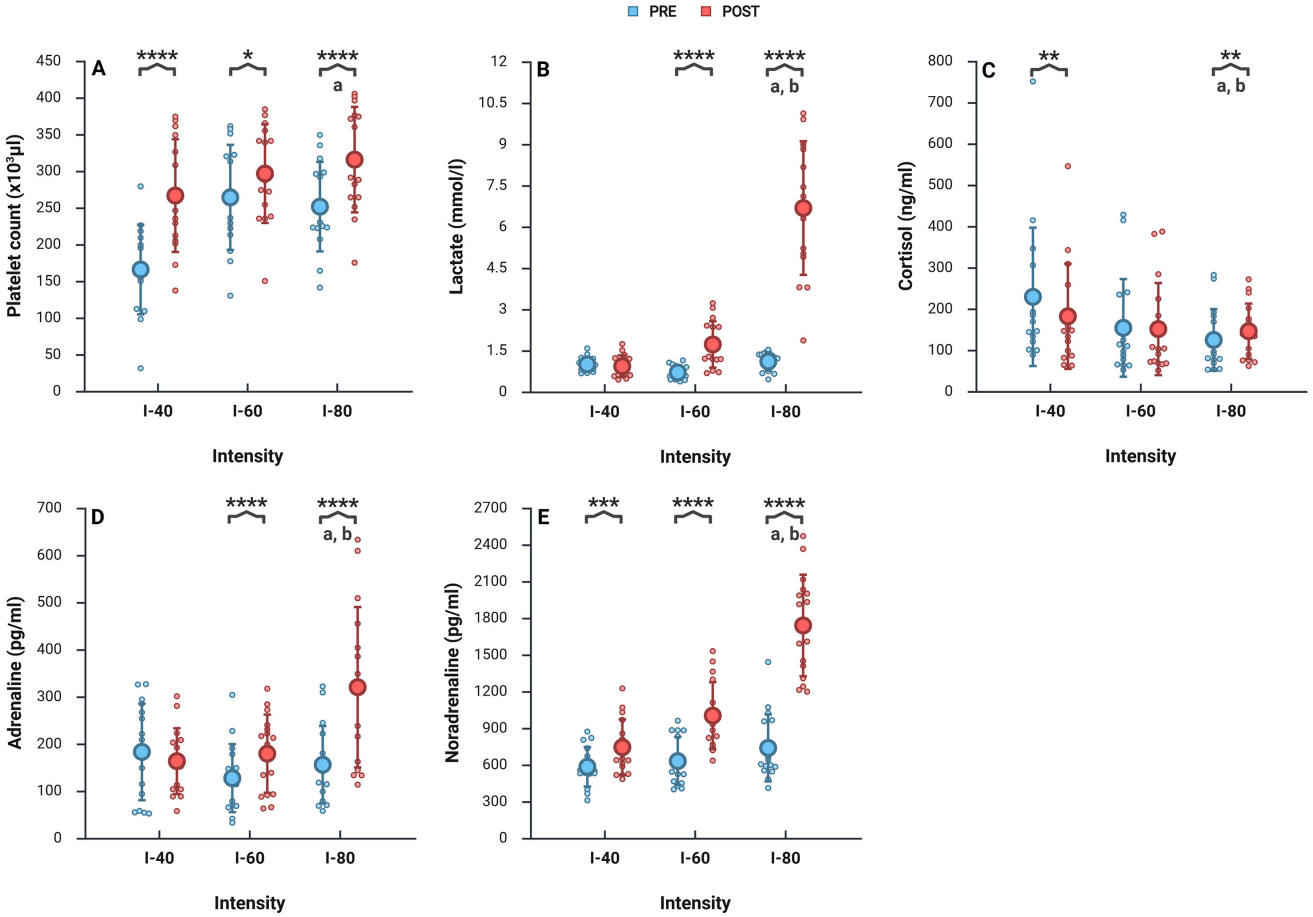
Effects of exercise intensity on platelet count, circulating lactate, cortisol, adrenaline and noradrenaline. Means (circles), standard deviations (vertical lines) and individual data points (small circles) of **(A)** platelet count (n = 15), **(B)** arterial lactate (n = 16), **(C)** arterial cortisol (n = 16), **(D)** arterial adrenaline (n = 16) and **(D)** arterial noradrenaline (n = 16). Data was analysed using a linear mixed effects model with two fixed effects: time [before (PRE) and after (POST) each intensity level] and intensity [cycling at 40% (I-40), at 60% (I-60) and at 80% (I-80) of individual VO_2_max]. * *p* < 0.05; ** *p* < 0.01; *** *p* < 0.001; **** *p* < 0.0001; a = significant difference from PRE-POST change at I-40; b = significant difference from PRE-POST change at I-60.

For platelet count (n = 15; **Figure 2A**), there was a *time × intensity* interaction [*F*(2,70.0) = 7.78, *p* = 0.001]. Increases in platelet count from PRE to POST were found at all intensity levels. Platelet count increased by ~61, 12 and 25% at I-40 (*p* < 0.001), I-60 (*p* = 0.015) and I-80 (*p* < 0.001), respectively. The increase at I-80 was smaller than at I-40 (*p* = 0.038) and showed a trend towards being greater than at I-60 (*p* = 0.072).

For log-transformed LAC-A (n = 16; **Figure 2B**), the results revealed *time × intensity* interaction [*F*(2,75.0) = 151.39, *p* < 0.001]. At I-40, no change in LAC-A was observed, but increases from PRE to POST were found at I-60 (~145%, *p* < 0.001) and I-80 (~507%, *p* < 0.001). The increase at I-80 differed from the non-significant change shown at I-40 (*p* < 0.001) and was greater than the increase shown at I-60 (*p* < 0.001).

For log-transformed CORT-A (n = 16; **Figure 2C**), a *time × intensity* interaction [*F*(2,75.0) = 8.69, *p* < 0.001] was observed. At I-40, a PRE-POST decrease (~21%, *p* = 0.005), whereas at I-80, a PRE-POST increase (~17%, *p* = 0.008) was shown. However, there was no change at I-60 (*p* = 0.824). The change at I-80 differed from that at both I-40 (*p* < 0.001) and I-60 (*p* = 0.041).

For log-transformed ADR-A (n = 16; **Figure 2D**), there was also a *time × intensity* interaction [*F*(2,75.0) = 21.45, *p* < 0.001]. At I-40, no change in ADR-A was observed. Instead, PRE–POST increases were shown at I-60 (~41%, *p* < 0.001) and I-80 (~106%, *p* < 0.001). The change at I-80 differed from the non-significant change shown at I-40 (*p* < 0.001) and was greater than at I-60 (*p* = 0.004).

For log-transformed NAD-A (n = 16; **Figure 2E**), a *time × intensity* interaction [*F*(2,75.0) = 26.31, *p* < 0.001] was also found. Increases in NAD-A from PRE to POST were observed at all intensities. The NAD-A increased by ~27, 59 and 136% at I-40 (*p* < 0.001), I-60 (*p* < 0.001) and I-80 (*p* < 0.001), respectively. The increase shown at I-80 was greater than at I-40 (*p* < 0.001) and I-60 (*p* < 0.001).

### 3.4 Predictors of changes in circulating BDNF and net cerebral lactate exchange

The results from the elastic net regression model are presented in **Table 2**. As exercise-induced changes in all investigated variables were the largest at I-80, data associated with the highest intensity were included in this model. There was a strong correlation between changes in ADR-A and NAD-A (*r* = 0.71, *p* = 0.003). To improve the interpretability of the model coefficients, ADR-A was excluded. The list of outcomes included changes at I-80 in mBDNF-S (ΔmBDNF-S_80_), proBDNF (ΔproBDNF_80_) and brain lactate exchange (ΔLAC-AV-B_80_). For ΔmBDNF-S_80_ and ΔproBDNF_80_, the list of predictors included PRE values at I-80 of platelet count (PLA_pre80_), LAC-A (LAC-A_pre80_), CORT-A (CORT-A_pre80_), NAD-A (NAD-A_pre80_) and fCSA%-II, as well as changes at I-80 in platelet count (ΔPLA_80_), LAC-A (ΔLAC-A_80_), CORT-A (ΔCORT-A_80_) and NAD-A (ΔNAD-A_80_). For ΔLAC-AV-B_80_, the list of predictors included ΔPLA_80_, ΔLAC-A_80_, ΔCORT-A_80_, ΔNAD-A_80_, ΔmBDNF-S_80_ and ΔproBDNF_80_.

**Table 2:** Results from Elastic Net regression model used to evaluate whether pre-exercise values at I-80 (pre80) of platelet count (PLA_pre80_), arterial lactate (LAC-A_pre80_), arterial cortisol (CORT-A_pre80_), arterial noradrenaline (NAD-A_pre80_) and/or percentage of muscle type II fCSA (fCSA%-II), as well as whether PRE-POST changes (Δ) at I-80 in platelet count (ΔPLA_80_), arterial lactate (ΔLAC-A_80_), arterial cortisol (ΔCORT-A_80_) and/or arterial noradrenaline (ΔNAD-A_80_) predict PRE-POST changes at I-80 in serum mature BDNF (ΔmBDNF-S_80_) and proBDNF (ΔproBDNF_80_). The same model was used to assess whether ΔPLA_80_, ΔLAC-A_80_, ΔCORT-A_80_, ΔNAD-A_80_, ΔmBDNF-S_80_ and/or ΔproBDNF_80_ can predict PRE-POST changes at I-80 in net cerebral lactate exchange (ΔLAC-AV-B_80_).

Outcome	Model performance		Predictors	*β*	π_j_
ΔmBDNF-S_80_					
(n = 14)	α	0.1	PLA_pre80_	0	0.653
	λ	2117.00	LAC-A_pre80_	0	0.546
	**R^2^**	**0.44**	**CORT-A_pre80_**	**-53.44**	**0.648**
	RMSE	1392.73	**NAD-A_pre80_**	**-129.73**	**0.632**
	MAE	1003.06	**fCSA%-II**	**138.12**	**0.739**
			**ΔPLA_80_**	**111.87**	**0.761**
			**ΔLAC-A_80_**	**148.43**	**0.617**
			ΔCORT-A_80_	0	0.468
			**ΔNAD-A_80_**	**482.21**	**0.935**
ΔproBDNF_80_					
(n = 14)	α	0.1	**PLA_pre80_**	**-25.00**	**0.816**
	λ	117.75	**LAC-A_pre80_**	**-20.53**	**0.826**
	**R^2^**	**0.54**	CORT-A_pre80_	0	0.709
	RMSE	92.47	**NAD-A_pre80_**	**-9.83**	**0.729**
	MAE	72.13	fCSA-II	0	0.705
			**ΔPLA_80_**	**23.87**	**0.890**
			**ΔLAC-A_80_**	**-9.88**	**0.678**
			**ΔCORT-A_80_**	**27.73**	**0.905**
			ΔNAD-A_80_	-1.30	0.595
ΔLAC-AV-B_80_					
(n = 13)	α	0.8	ΔPLA_80_	0	0.512
	λ	0.04	**ΔLAC-A_80_**	**0.03**	**0.762**
	**R^2^**	**0.28**	ΔCORT-A_80_	0	0.347
	RMSE	0.08	ΔNAD-A_80_	0	0.388
	MAE	0.06	ΔmBDNF-S_80_	0	0.459
			ΔproBDNF_80_	0	0.463

n, sample size; α, optimal hyperparametre for the Elastic Net Leave-One-Out Cross-Validation model, representing mixing ratio between L1 (lasso) and L2 (ridge) penalties; λ, optimal hyperparametre for the model, controlling overall strength of the regularization of the model coefficients; R^2^, coefficient of determination, proportion of variance in the outcome explained by the model; RMSE, Root Mean Square Error of the model, average magnitude of prediction error; MAE, Mean Absolute Error of the model; *β*, coefficient estimate; π_j_, selection stability, defined as the proportion of 1000 bootstrap samples in which predictor had a non-zero coefficient.

Values in bold indicate meaningful contribution of the respective predictor to the model.

For ΔmBDNF-S_80_ (n = 14), the model (with an optimal α = 0.1 and λ = 2117.00) demonstrated moderate predictive power (*R²* = 0.44, RMSE = 1392.73, MAE = 1003.06). Among the predictors, the ΔNAD-A_80_ showed the largest positive and stable association with ΔmBDNF-S_80_ (*β* = 482.21, π_j_ = 0.935). Positive and moderately stable associations with the outcome were also seen for fCSA%-II (*β* = 138.12, π_j_ = 0.739), ∆PLA_80_ (*β* = 111.87, π_j_ = 0.761) and ∆LAC-A_80_ (*β* = 148.43, π_j_ = 0.617). However, stable but negative associations with ΔmBDNF-S_80_ were shown for CORT-A_pre80_ (*β* = -53.44, π_j_ = 0.648) and NAD-A_pre80_ (*β* = -129.73, π_j_ = 0.632). The PLA_pre80_, LAC-A_pre80_ and ∆CORT-A_80_ showed no meaningful contribution to the model (*β* = 0).

For ΔproBDNF_80_ (n = 14), the model (with an optimal α = 0.1 and λ = 117.75) showed a moderate-to-strong fit (*R²* = 0.54, RMSE = 92.47, MAE = 72.13). Positive and stable associations with ΔproBDNF_80_ were observed for ∆PLA_80_ (*β* = 23.87, π_j_ = 0.890) and ∆CORT-A_80_ (*β* = 27.73, π_j_ = 0.905). However, negative and stable associations with the outcome were observed for PLA_pre80_ (*β* = -25.00, π_j_ = 0.816), LAC-A_pre80_ (*β* = -20.53, π_j_ = 0.826), NAD-A_pre80_ (*β* = -9.83, π_j_ = 0.729) and ∆LAC-A_80_ (*β* = -9.88, π_j_ = 0.678). The CORT-A_pre80_, ΔNAD-A_80_ and fCSA%-II did not contribute meaningfully to the model (*β* = 0).

Finally, for ΔLAC-AV-B_80_ (n = 13), the model (with an optimal α = 0.8 and λ = 0.04) demonstrated moderate power (*R²* = 0.28, RMSE = 0.08, MAE = 0.06). A positive and stable association with ΔLAC-AV-B_80_ was shown only for ∆LAC-A_80_ (*β* = 0.03, π_j_ = 0.762). The ∆PLA_80_, ∆CORT-A_80_, ΔNAD-A_80_, ΔmBDNF-S_80_ and ΔproBDNF_80_ were not retained in the model (*β* = 0).

### 3.5 Exploratory analysis of BDNF Val66Met polymorphism influence on changes in BDNF and net cerebral lactate exchange

The BDNF Val66Met polymorphism was expressed in 5 out of 16 participants, while the remaining 11 were Val66Val carriers (**Table 1**). For mBDNF-S (n = 16; **Supplementary Figure 1A**), neither a main effect nor an interaction with BDNF genotype was shown. For the log-transformed forearm venous proBDNF (n = 14; **Supplementary Figure 1B**), no interaction with BDNF genotype was observed. Instead, there was a main effect of BDNF genotype [*F*(1,13.0) = 6.97, *p* = 0.020], with higher concentrations in Val66Val carriers shown both PRE and POST exercise across all exercise intensities. For LAC-AV-B (n = 16; **Supplementary Figure 1C**), there was a main effect of BDNF genotype [*F*(1,14.0) = 5.86, *p* = 0.030], and an indicated tendency of a *time × BDNF genotype* interaction [*F*(1,70.0) = 3.50, *p* = 0.066].

### 3.6 Association between muscle release of lactate and muscle release of proBDNF

No correlation was shown between previously reported muscle release of proBDNF (Tarassova et al., 2025) and muscle release of lactate (*r* = -0.09, *p* = 0.763). This result will support the interpretation of the main findings discussed below.

## 4 Discussion

This study aimed to identify correlates of exercise-induced changes in circulating BDNF isoforms and in net cerebral lactate exchange. Exercise-induced increases in mBDNF-S were greater in participants with a higher fCSA%-II and were positively associated with changes in platelet count, noradrenaline and lactate, but not cortisol. In contrast, increases in proBDNF were positively associated with platelet count but negatively associated with pre-exercise noradrenaline and lactate, as well as with lactate responses. ProBDNF responses were also positively related to changes in cortisol and were not predicted by muscle fibre type. Increases in net cerebral lactate uptake were observed after high-intensity exercise but not after low- or moderate-intensity exercise, and they were related only to the magnitude of the circulating lactate increase. Finally, exploratory analyses of the moderating effect of BDNF genotype revealed a possible pattern whereby proBDNF levels may be higher in Val66Val carriers, while increases in net cerebral lactate uptake may be greater in Val66Met carriers. These observations merit further investigation in larger cohorts.

### 4.1 Effects of exercise intensity

We previously reported exercise-intensity-dependent effects of a single bout of cycling for platelet count, mBDNF-S and proBDNF (Tarassova et al., 2025). In the current study, based on the same experiment, we additionally show an increased net cerebral lactate uptake after cycling at 80% VO₂max (**Figure 1C**) as well as exercise-intensity dependent effects for arterial lactate (**Figure 2B**), adrenaline and noradrenaline (**Figures 2D, E**), with the greatest increases shown after exercise at 80% VO₂max. These results are in line with an earlier report (Hashimoto et al., 2018), confirming that high-intensity exercise stimulates greater availability of BDNF, platelet count, lactate and catecholamines in the peripheral circulation. Furthermore, our results suggest that high-intensity, but not low- or moderate-intensity, exercise increases net cerebral lactate uptake. The observed net cerebral lactate uptake is in line with previously proposed lactate shuttle between active skeletal muscle and the brain, driven by an increased cerebral energy demand during exercise (Dalsgaard et al., 2002; van Hall et al., 2009). However, whether elevated brain lactate, as suggested in animal models (El Hayek et al., 2019; Moberg et al., 2024), stimulates the regulation of exercise-induced BDNF production and release locally in the brain remains uncertain in humans. Notably, in the same dataset (Tarassova et al., 2025), neither mBDNF uptake nor its release across the brain was observed.

Changes in arterial cortisol observed in this study (**Figure 2C**) mainly followed the earlier proposed circadian fluctuation (Begliuomini et al., 2008), with the highest peak at baseline in the morning. Interestingly, the authors in Hill et al. (2008) noted that, after correction for circadian fluctuations and plasma volume reduction, cortisol levels decreased after cycling at 40% VO₂max. Our results show a ~21% decrease in arterial cortisol without such corrections. Nevertheless, arterial cortisol increased slightly (~17%) but significantly after exercise at 80% VO₂max. This indicates a sufficient exercise-induced activation of the HPA axis. However, whether it facilitated the previously suggested trophic influence of cortisol in the brain, inducing neuroplastic effects (Gray et al., 2013; Tsimpolis et al., 2024), remains uncertain.

### 4.2 Predictors of exercise-induced changes in BDNF isoforms

#### 4.2.1 Association with platelet count, noradrenaline and cortisol

Changes in platelet count and noradrenaline appear to be the main correlates of exercise-induced changes in mBDNF-S (**Table 2**). Although the associations do not necessarily indicate a causal relationship, these findings may suggest catecholamine-mediated splenic platelet release of mBDNF. While platelets contain mBDNF (Le Blanc et al., 2020) and are proposed to be the main driver of exercise-induced increases in mBDNF-S (Walsh and Tschakovsky, 2018; Tarassova et al., 2025), increases in catecholamines can induce splenic contraction leading to platelet release into the circulation (Wadenvik and Kutti, 1987). It has also been shown that some, though not all, platelets can express proBDNF as well (Le Blanc et al., 2020). Furthermore, as platelets express several proteases that can facilitate BDNF maturation (Blakytny et al., 2004; Whiteheart, 2011), Le Blanc et al. (2020) suggested that cleavage of proBDNF into mBDNF, might occur inside the platelets. However, platelet activation with several agonists did not result in detectable proBDNF release or changes in intraplatelet proBDNF levels (Le Blanc et al., 2020). This study did not assess exercise-induced platelet activation. Notably, Leiter and Walker (2019) proposed that physical exercise may represent a distinct form of platelet activation, as classical activation markers were not detected in the platelet proteomic profile following exercise in mice (Leiter et al., 2019). Hence, the association between platelet count and plasma proBDNF observed in the present study may still suggest an exercise-induced release of proBDNF from platelets into the circulation.

Hormonal activity of noradrenaline has also been suggested to stimulate neuroplastic signalling through the previously proposed link between exercise-induced changes in noradrenaline and brain BDNF (Ji et al., 2005; McMorris, 2016). Cortisol, as well as its interaction with central BDNF via TrkB activation, has also been implicated in the regulation of neuroplasticity (Gray et al., 2013; Tsimpolis et al., 2024). In the present study, larger increases in noradrenaline were positively associated with larger increases in mBDNF-S (**Table 2**), suggesting the potential contribution of exercise-induced catecholaminergic activity to increases in circulating mBDNF. Furthermore, consistent with previous reports (Gustafsson et al., 2009; Röja et al., 2025), no association between changes in cortisol and mBDNF-S was shown. In contrast, an association between changes in proBDNF and noradrenaline was not observed. Instead, larger increases in cortisol were associated with larger increases in proBDNF. While mBDNF has previously been linked to synaptic strengthening and hippocampal long-term potentiation (Woo et al., 2005), processes important for learning and memory (Sweatt, 2016), proBDNF has been linked to synaptic pruning processes relevant for hippocampal plasticity (Woo et al., 2005). Therefore, catecholamine-related increases in mBDNF-S and cortisol-related increases in proBDNF observed in the present study may suggest the potential link to mechanisms previously associated with synaptic strengthening and pruning, respectively. However, whether the relationship between these exercise factors is causal warrants further investigation, as the present study demonstrates only associations. Moreover, our recent report suggests that exercise-induced increases in circulating BDNF isoforms may primarily reflect peripheral sources rather than brain secretion (Tarassova et al., 2025). Thus, our present data cannot aid in the interpretation of mBDNF-noradrenaline and proBDNF-cortisol interactions in CNS modulation in relation to exercise.

Previous literature also suggests a negative impact of psychological stress on BDNF expression and trafficking (Tsimpolis et al., 2024). In the present study, higher pre-exercise cortisol and noradrenaline levels predicted dampened increases in mBDNF-S (**Table 2**). Even though pre-exercise cortisol did not appear to predict changes in proBDNF, an inverse association between changes in proBDNF and pre-exercise noradrenaline was also observed. These findings suggest a possible association between elevated stress markers before exercise, possibly due to anticipation of heavy exercise, and reduced platelet-driven increase in mBDNF-S, as well as dampened increase in proBDNF.

#### 4.2.2 Association with blood lactate levels

In line with an earlier report (Ferris et al., 2007), our results indicated a positive association between exercise-induced increases in mBDNF-S and circulating lactate (**Table 2**). This may suggest a potential contribution of elevated blood lactate, possibly derived from active skeletal muscle, to the increase in mBDNF-S. In line with this, Schiffer et al. (2011) reported that venous sodium lactate infusion at rest in humans, producing a higher peak plasma lactate (~13 mmol·l⁻¹) than observed after high-intensity exercise in the present study (~7 mmol·l⁻¹), was associated with a rapid increase in mBDNF. However, exercise-induced increases in platelet-bound mBDNF have been suggested (Walsh and Tschakovsky, 2018) and shown (Tarassova et al., 2025) to be fully explained by exercise-induced augmentation in circulating platelet levels, likely driven by splenic platelet release. Moreover, in the study by Schiffer et al. (2011), platelet count remained unchanged after lactate infusion. Still, although the direct link between elevated blood lactate and splenic contraction remains largely unknown, previous evidence suggests that platelet activation may stimulate lactate production and glucose uptake in human platelets (Nayak et al., 2021). Another plausible explanation could be that the association between increases in blood lactate levels and mBDNF-S observed in the present study simply reflects a parallel effect of catecholamines on these exerkines. Existing evidence appears to support the latter speculation as, besides stimulation of splenic contraction (Wadenvik and Kutti, 1987), exercise-induced increases in adrenaline also stimulate increased lactate accumulation (Hamann et al., 2001; Watt et al., 2001).

A small but significant increase in muscle release of proBDNF after high-intensity exercise was previously reported in the same group of healthy adults (Tarassova et al., 2025). Here, as muscle lactate release occurred at the same intensity, this may suggest a lactate-driven release of proBDNF. However, this interpretation remains uncertain, as muscle lactate release was not correlated with proBDNF release (*r* = −0.09, *p* = 0.763). Our results also advocate that higher pre-exercise arterial lactate levels predict smaller exercise-induced increases in proBDNF after cycling at 80% VO₂max, and that larger increases in lactate were negatively associated with changes in proBDNF (**Table 2**). This might indicate an inverse relationship between changes in lactate and proBDNF, which could be related to the type I muscle fibre-dominant expression of proBDNF, as type I muscle fibres are predominantly lactate consumers and not producers. It should be noted that the substantial individual differences in proBDNF changes observed in our study (**Figure 1B**) warrant caution in results interpretation.

#### 4.2.3 Association with muscle fibre type

Previous literature suggests greater recruitment of fast-twitch, type II muscle fibres, during high-intensity exercise compared to lower intensities (Vøllestad et al., 1984; Sale, 1987; MacInnis and Gibala, 2017). It has also been shown that simultaneous elevation of lactate, predominantly produced by type II muscle fibres (Gladden, 2004; Brooks et al., 2022), and circulating catecholamines can improve contractility and counteract fatigue in fast-twitch muscles caused by hyperkalaemia during intensive exercise (Hansen et al., 2005). Although mBDNF is not detectable in human skeletal muscle (Edman et al., 2024), in the present study, exercise-induced increases in mBDNF-S were positively associated with fCSA%-II and were paralleled with increases in lactate and catecholamines (**Figures 2B, D–E**; **Table 2**). Thus, our results may further support catecholamine-driven elevation of mBDNF-S, suggesting that, in individuals with higher fCSA%-II, high-intensity exercise may indirectly facilitate greater availability of mBDNF-S.

The reason for the non-significant association between exercise-induced changes in circulating proBDNF and fCSA% in the present study (**Table 2**) is, however, unclear. A negative association might be expected, as in humans, a positive correlation between muscle proBDNF expression and type I muscle fCSA% was previously observed (Edman et al., 2024; Röja et al., 2025). Instead, our findings suggest that exercise-induced changes in circulating proBDNF are either independent of muscle fibre type or influenced by the dynamics of proBDNF release from active muscle and its cleavage to the mature form during exercise (Kojima et al., 2019). This warrants further investigation in larger cohorts.

### 4.3 Predictors of net cerebral lactate exchange

In the present study, increased net cerebral lactate uptake was observed after cycling at 80% VO₂max (**Figure 1C**) and was predicted by arterial lactate concentrations (**Table 2**). Lactate has been proposed to stimulate BDNF expression in the brain in exercising mice (El Hayek et al., 2019; Hwang et al., 2023), suggesting that increased net cerebral lactate exchange during exercise may contribute to activity-dependent neurotrophic signalling. However, the present findings do not suggest that these processes are reflected in circulating BDNF levels, at least not in the acute phase after exercise (**Table 2**). Still, adrenergic activity may indirectly influence cerebral lactate uptake by modulating whole-body glycolysis (Rasmussen et al., 2011).

### 4.4 Exploratory analysis of BDNF genotype influence on BDNF isoforms and net cerebral lactate exchange

Although the sample size was insufficient for genotype analyses, we still have also explored the potential influence of Val66Met polymorphism on exercise-induced changes in mBDNF-S, proBDNF and net cerebral lactate exchange. In line with Ashcroft et al. (2025), BDNF genotype did not moderate the effect of exercise on mBDNF-S in the present study (**Supplementary Figure 1A**). Substitution of amino-acid valine to methionine at codon 66 (Val66Met) occurs in the pro-domain of the BDNF protein, and after proBDNF cleavage to mBDNF, it is unlikely that this mutation would affect mBDNF intrinsic activity, as this mutation is no longer present on the mature protein. While previous reports found no difference in proBDNF levels between Val66Val and Val66Met groups after cycling exercise (Piepmeier et al., 2020) or 1-h lactate infusion (Röja et al., 2025), Val66Val carriers in the present study showed higher proBDNF levels before and after exercise across all intensities (**Supplementary Figure 1B**). In contrast, our results showed higher levels and a tendency of larger exercise-induced increases in net cerebral lactate uptake in Val66Met carriers, irrespective of intensity (**Supplementary Figure 1C**). This observation suggest that Val66Met polymorphism may reduce proBDNF secretion, as previously suggested (Egan et al., 2003), and that Val66Met carries may be more dependent on lactate supply to meet an increased cerebral energy demand during exercise and to benefit brain function. However, given an uneven distribution between Val66Met and Val66Val carriers (n = 5 vs. n = 11) and a very small sample size in the Val66Met group (n = 5), the present study results should be regarded as hypothesis-generating and require validation in larger cohorts.

## 5 Methodological considerations

### 5.1 Strengths

The present study included data from a well-characterized population of well-trained, physically fit adults, previously described in detail in Tarassova et al. (2025). We have investigated the effects of 20 min of cycling ergometre exercise at three intensities (light, moderate, and high) on lactate levels, including lactate exchange between the brain and skeletal muscle. Furthermore, the effect of exercise on several endocrine markers of stress, such as cortisol, adrenaline and noradrenaline, reflecting the neuroendocrine pathways of both the sympathetic–adrenal–medullary system and hypothalamic–pituitary–adrenal (HPA) axis, was also assessed. The analysed associations of mBDNF, proBDNF, and net cerebral lactate exchange with platelet count, arterial lactate and several biomarkers of stress enabled further exploration of the mechanisms driving increases in BDNF isoforms and net cerebral lactate exchange after a single bout of aerobic exercise.

### 5.2 Limitations

This study used blood samples collected according to the data-collection protocol described in our previous report (Tarassova et al., 2025). Hence, the authors refer the reader to the well-described major limitations in Tarassova et al. (2025). Additional limitations in the present study include the analysis of arterial cortisol levels from morning samples. Cortisol levels follow a circadian rhythm, peaking after awakening (Begliuomini et al., 2008). Moreover, cycling intensities were performed in an increasing order, which may induce cumulative effects, such as increased fatigue, lactate and catecholamine accumulation. However, the standardized 30 min of rest between intensities may have reduced the latter effect. From our observations, the cumulative effects on fatigue and lactate accumulation are negligible but might be evident, e.g., in hormonal responses and platelet count. The 30 min of recovery did not seem sufficient for the platelet count. Baseline platelet count measured before moderate- and high-intensity cycling remained elevated at the level observed after cycling at 40% of VO₂max. The latter may have reduced the platelet readily releasable pool, potentially explaining the smaller increases observed after cycling at 60% and 80% of VO₂max, compared to the increase shown after the first cycling bout. The sample size for the analysis of potential moderation effects of the BDNF genotype on exercise-induced changes in BDNF isoforms and net cerebral lactate exchange was also insufficient. Consequently, cautious interpretation of these results is warranted and requires validation in larger cohorts.

## 6 Conclusion

Levels of mBDNF-S, proBDNF, platelet count, adrenaline, noradrenaline, and lactate all increased following exercise in an intensity-dependent manner, while cortisol increase was shown only after exercise at 80% VO₂max. Exercise-induced increases in mBDNF-S were positively associated with increases in platelet count and noradrenaline. These associations are consistent with a model in which exercise-induced neuroendocrine activation promotes splenic contraction and platelet release, thereby increasing circulating BDNF. In contrast, while exercise-induced increases in proBDNF were also related to increased platelet count, the increase in proBDNF was predicted by larger increases in cortisol, but not in noradrenaline. Net cerebral lactate uptake increased after high-intensity exercise and was predicted by arterial lactate concentrations but was not associated with circulating BDNF. Finally, the present study characterizes associations between exercise-induced changes in circulating BDNF isoforms, neuroendocrine responses, and net cerebral lactate exchange, providing insight into physiological mechanisms that may contribute to exercise-induced neuroplasticity and merit further investigation.

## Data Availability

The raw data supporting the conclusions of this article will be made available by the authors, without undue reservation.
