# Supplementary material for: Physiological predictors of exercise-induced increases in circulating BDNF isoforms and net cerebral lactate exchange in healthy adults
Source: Front Physiol. 2026 Jul 6;17:1862716. doi: 10.3389/fphys.2026.1862716 (PMC13381295; doi:10.3389/fphys.2026.1862716)
Supplement: Supplementary file 1 [file Supplementaryfile1.docx]

Supplementary Material

# Supplementary Figures


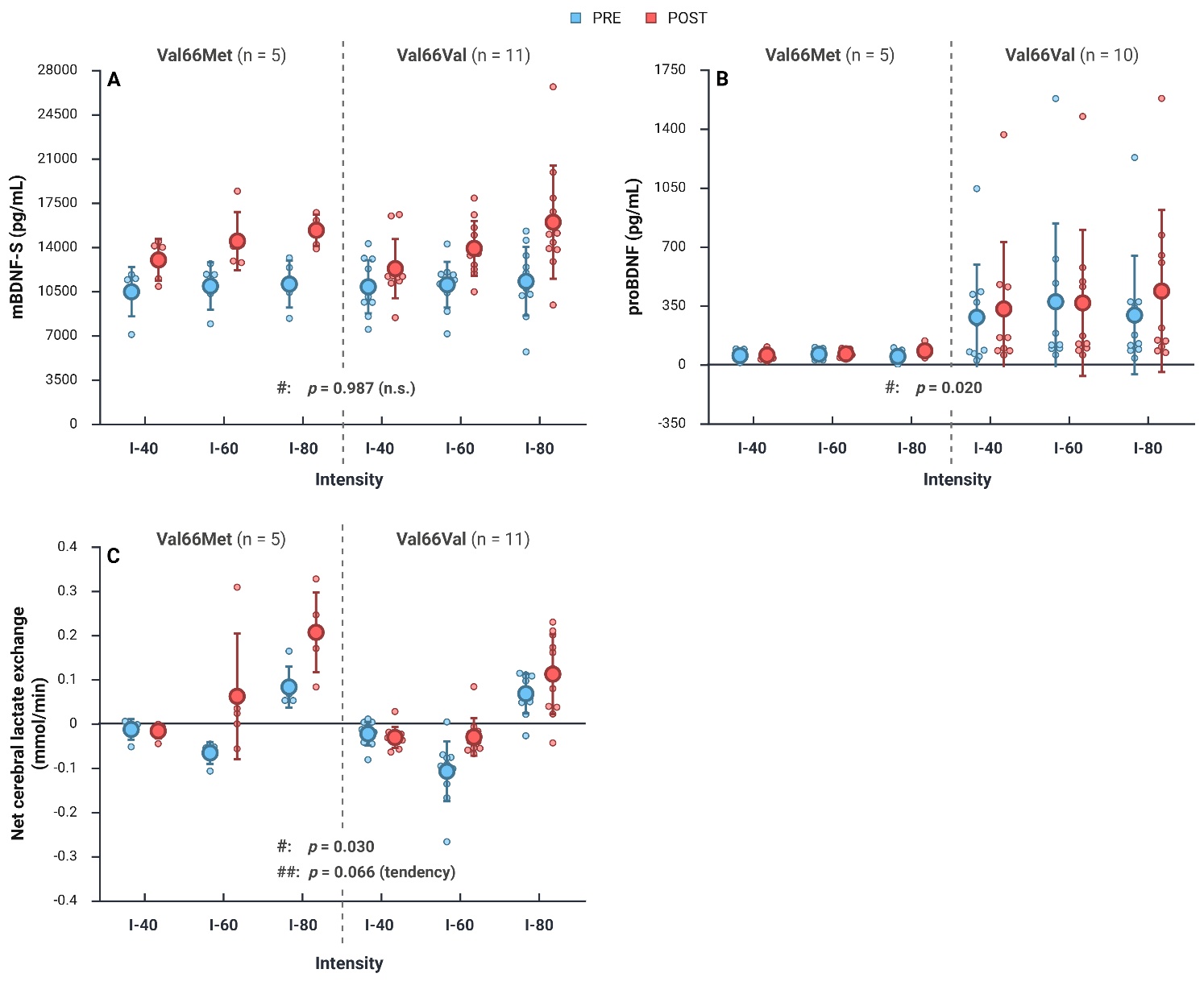


**Supplementary Figure 1.** **Influence of BDNF genotype on exercise-induced changes in forearm venous mBDNF-S, proBDNF and net cerebral lactate exchange.** Means (circles), standard deviations (vertical lines) and individual data points (small circles) of (**A**) forearm venous mBDNF-S (n = 16), (**B**) forearm venous proBDNF (n = 14) and (**C**) net cerebral lactate exchange. Data was analysed using a linear mixed effects model with three fixed effects: time [before (PRE) and after (POST) each intensity level], intensity [cycling at 40% (I-40), at 60% (I-60) and at 80% (I-80) of individual VO_2_max] and BDNF genotype [Val66Met and Val66Val]. **#** = main effect of BDNF genotype; **##** = *time* × *BDNF genotype* interaction.
